# Treatment of posterior circulation stroke: Acute management and secondary prevention

**DOI:** 10.1177/17474930221107500

**Published:** 2022-06-28

**Authors:** Hugh S Markus, Patrik Michel

**Affiliations:** 1 Department of Clinical Neurosciences, University of Cambridge, Cambridge, UK; 2 Stroke Center, Neurology Service, Department of Clinical Neurosciences, Lausanne University Hospital and University of Lausanne, Lausanne, Switzerland

**Keywords:** Vertebral, basilar, posterior circulation, vertebrobasilar, stenting, acute stroke therapy, treatment, prevention

## Abstract

One-fifth of strokes occur in the territory of the posterior circulation, but their management, particularly acute reperfusion therapy and neurointervention procedures for secondary prevention, has received much less attention than similar interventions for the anterior circulation. In this review, we overview the treatment of posterior circulation stroke, including both interventions in the acute setting and secondary prevention. We focus on areas in which the management of posterior circulation stroke differs from that of stroke in general and highlight recent advances.

Effectiveness of acute revascularization of posterior circulation strokes remains in large parts unproven. Thrombolysis seems to have similar benefits and lower hemorrhage risks than in the anterior circulation. The recent ATTENTION and BAOCHE trials have demonstrated that thrombectomy benefits strokes with basilar artery occlusion, but its effect on other posterior occlusion sites remains uncertain. Ischemic and hemorrhagic space-occupying cerebellar strokes can benefit from decompressive craniectomy.

Secondary prevention of posterior circulation strokes includes aggressive treatment of cerebrovascular risk factors with both drugs and lifestyle interventions and short-term dual anti-platelet therapy. Randomized controlled trial (RCT) data suggest basilar artery stenosis is better treated with medical therapy than stenting, which has a high peri-procedural risk. Limited data from RCTs in stenting for vertebral stenosis suggest that intracranial stenosis is currently best treated with medical therapy alone; the situation for extracranial stenosis is less clear where stenting for symptomatic stenosis is an option, particularly for recurrent symptoms; larger RCTs are required in this area.

## Introduction

Stroke is globally the second leading cause of death and the third cause of death and disability.^1^ One-fifth of strokes occur in the vertebrobasilar territory (also known as posterior) circulation.^2^ Diagnosis of posterior circulation stroke and transient ischemic attack (TIA) can be more challenging than anterior circulation syndromes, and widely used screening protocols such as the face-arm-speech test (FAST) are less sensitive.^3^ Optimal management of posterior circulation stroke, particularly acute reperfusion therapy and neurointervention procedures for secondary prevention, has received much less attention than similar interventions for the anterior circulation.^3^ However, recent research and ongoing studies are improving our understanding. In this review, we cover the treatment of posterior circulation stroke, covering both interventions in the acute setting and secondary prevention. We focus on areas in which management of posterior circulation stroke differs from that of stroke in general and highlight recent advances.

## Methods

The authors conducted a literature search on PubMed from its inception to 29 March 2022. Only English-language articles were reviewed and included. All searches included the terms “posterior circulation,” “brainstem,” “cerebellar,” “occipital,” “thalamic,” “basilar,” “vertebral,” and “posterior cerebral.” We added the following terms with an “AND” function: for acute revascularization: “thrombolysis,” “alteplase,” “rtPA,” “endovascular treatment,” “thrombectomy,” “stentretriever,” “thromboaspiration”; “haemorrhage” and “haemorrhagic transformation”; for decompressive craniectomy: “mass effect,” “space occupying,” “edema” “decompressive/decompression,” “craniectomy,” “drainage,” and “ventriculostomy”; for posterior fossa hemorrhage: “haemorrhage”; and for secondary prevention: “antiplatelet,” “intensive,” “angioplasty,” and “stenting.” Finally, the following filters were applied during the search and again when manually selecting articles: “case-control study,” “cohort study,” “comparative analysis,” “randomized trial,” “meta-analysis,” “subgroup analysis,” and “pooled analysis.” In the retrieved articles, the reference lists were checked for further studies.

## Acute management

### Intravenous thrombolysis

We found no RCTs comparing intravenous thrombolysis (IVT) with antithrombotic treatment for the posterior circulation alone. The International Stroke Trial-3 was one of the only IVT RCT where the site of stroke was recorded; in the pre-specified subgroup analysis, posterior circulation strokes had benefits similar to anterior circulation strokes.^4^

Retrospective analyses, despite their significant limitations, have shown similar results. Long-term outcomes after IVT seem to be at least as favorable in the posterior as in the anterior circulation (relative risk (RR): 1.19; 95% confidence interval (CI), 1.06–1.33 for mRS 0–2), and the risk of symptomatic intracranial hemorrhage (ICH) is about half (RR: 0.49, 0.32–0.75).^5^ Another meta-analysis suggested a more frequent favorable outcome (odds ratio (OR): 1.36, 1.08–1.71) and confirmed lower rates of ICH (OR: 0.32, 0.21–0.49).^6^

A specific concern is the optimal treatment for basilar artery occlusion (BAO), which can have a devastating clinical outcome. A case series of 116 BAO patients showed that distal clot location was more likely to recanalize with IVT and that recanalization was associated with survival and improved outcome.^7^ In the BASICS (Basilar Artery International Cooperation Study) registry including 121/592 (20.4%) patients receiving IVT, this treatment was not superior to antithrombotic treatment (adjusted RR: 0.94, 0.60–1.45 for poor outcome) in mild-to-moderate strokes;^8^ there was a possibility of better outcome in severe strokes (adjusted RR: 0.88, 0.76–1.01 for poor outcome). In a retrospective analysis of 110 patients with BAO undergoing IVT before thrombectomy, IV tenecteplase led to a higher rate of radiological reperfusion than IV alteplase,^9^ and an RCT comparing these two drugs before endovascular treatment (EVT) is underway (Post-Eternal: https://clinicaltrials.gov/ct2/show/NCT04454788).

In conclusion, thrombolysis for posterior circulation stroke appears to have similar efficacy as for the anterior circulation, with lower hemorrhage risk. It may be the treatment of choice in certain posterior circulation strokes, including distal posterior cerebral artery (PCA) occlusion, and minor strokes. IVT does not preclude subsequent endovascular therapy although the benefit of bridging remains unproven in the posterior circulation.

### Acute endovascular revascularization

EVT in the posterior circulation, in particular mechanical thrombectomy, is most often performed and best studied for BAO. There are only a few observational studies for PCA occlusions, and no systematic analyses of acute EVT in vertebral artery (VA) occlusions.

Adjusted retrospective comparisons of EVT in the posterior circulation as a whole show similar clinical effectiveness to the anterior circulation in two large studies^10,11^ but worse outcome in another.^12^ Rates of symptomatic ICH are similar to those in the anterior circulation.

### Endovascular treatment of BAO

Until very recently, there was no convincing data that endovascular treatment for BAO was more effective than thrombolysis. In the non-randomized prospective international BASICS registry,^8^ 288 (48.6%) of 592 patients underwent EVT. In mild-to-moderately severe stroke, EVT was associated with worse 3-month outcome (mRS score of ⩽3) when compared to IVT (adjusted RR: 1.49, 1.00–2.23 for poor outcome), and no difference was seen in severe strokes (adjusted RR: 1.06, 0.91–1.22). Rates of symptomatic ICH were higher with EVT than with thrombolysis. In contrast, the non-randomized prospective Chinese BASILAR cohort showed a favorable 3-month shift in mRS when compared to standard medical treatment (adjusted OR: 3.08, 2.09–4.55).^13^ Symptomatic ICH was increased with EVT (7.1% vs 0.5%, *p* < 0.001).

**Figure 1.**
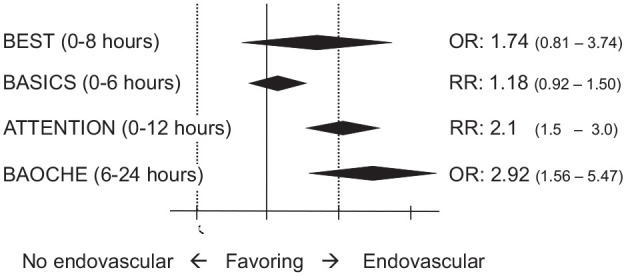
Results of the four large randomized controlled trials of endovascular treatment in patients with acute stroke from basilar artery occlusion.^14,15^ The odds or risk ratios and 95% confidence intervals are shown for a favorable outcome defined as modified Rankin score of 0–3 at 3 months. BEST: Basilar artery occlusion Endovascular intervention versus Standard medical Treatment;^14^ BASICS: BASilar artery International Cooperation Study;^15^ ATTENTION: EndovAscular TreaTmENT for acute basilar artery occlusION;^17^ BAOCHE: Basilar Artery Occlusion CHinese Endovascular trial;^18^ OR: odds ratio; RR: risk ratio.

The randomized controlled Chinese BEST trial (Basilar artery occlusion Endovascular intervention versus Standard medical Treatment) was stopped after 131 patients due to a 22% crossover rate.^14^ In the intention-to-treat analysis, it failed to show superiority of EVT over standard medical treatment (with a 32% IVT rate): the adjusted OR for favorable outcome (modified Rankin 0–3 at 3 months) was 1.74 (95% CI: 0.81–3.74) (Figure 1). Statistical significance likely has been lost because of the high cross-over rate to EVT.

**Figure 2.**
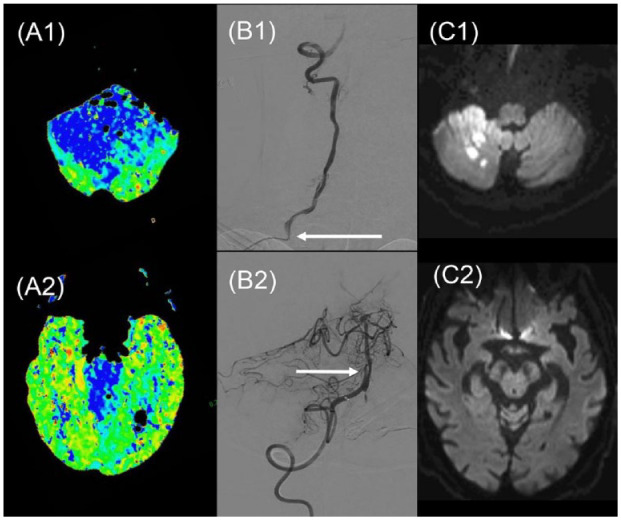
87-year-old man with acute BAO, NIHSS = 8, randomized to endovascular treatment in the BASICS trial:^15^ (a) extensive hypoperfusion (blue color) on mean transit time on perfusion CT from the medullary (A1) to midbrain (A2) levels. Thrombolysis at 130 min. (b) Conventional angiography showing tight stenosis at right vertebral origin (B1: arrow) and basilar artery occlusion (B1, top of image); thrombectomy with basilar artery recanalization at 4.2 h after onset, complicated by a non-stenosing mid-basilar dissection (B2, arrow). (c) Sub-acute diffusion-weighted MRI showing limited stroke volume in the right cerebellum (C1) and no visible lesion in the midbrain (C2). Favorable outcome at 3 months with minimal disability. Copyright Patrik Michel.

The international BASICS RCT with 300 patients showed no benefit of EVT over best medical treatment (with a 79% IVT rate), with a risk ratio for favorable functional outcome at 3 months of 1.18 (95% CI: 0.92–1.50) (Figure 1).^15^ In both BEST and BASICS, the symptomatic ICH rates were non-significantly higher after EVT. Figure 2 shows a patient randomized in the BASICS trial with extensive posterior fossa hypoperfusion, successfully recanalized with thrombectomy.

However, two recent trials presented at the European Stroke Conference 2022, after our systematic review was performed, showed convincing data on the effectiveness of EVT for BAO.^16^ ATTENTION (EndovAscular TreaTmENT for acute basilar artery occlusION) recruited patients within 0–12 h from the estimated time of stroke onset in China.^17^ A total of 340 patients were randomly assigned to thrombectomy or best medical management in a 2:1 ratio. There was a highly significant improvement in the primary endpoint of modified Rankin score 0–3 at 90 days which was achieved in 104/226 (46%) of the endovascular therapy group and 26/114 (22.8%) of the best medical management group; adjusted risk ratio of 2.1 (95% CI: 1.5–3.0).^16^ About one-third of patients received IVT. There was a trend to more symptomatic ICH with thrombectomy, but also to less mortality at 90 days.

BAOCHE (Basilar Artery Occlusion CHinese Endovascular trial) differed in that it recruited patients within 6 to 24 h of symptom onset where the patient was ineligible for IV thrombolysis or had received IVT without recanalization.^18^ The planned sample size was 318, but after a planned interim analysis after 212 patients, the data and safety monitoring committee recommended early termination of the trial due to highly significant differences between the two treatments. In 217 patients available for the final analysis, 51 of 110 (46.4%) randomized to thrombectomy achieved mRS 0–3 at 90 days, compared with 26/107 (24.3%) receiving best medical therapy, giving an adjusted OR of improved outcome of 2.92 (95% CI: 1.56–5.47).^16^ Despite an increase of symptomatic ICH and early mortality with ICH, this was the first EVT trial to demonstrate a reduction of 90-day mortality.

It is not yet known whether these two trials confirm the absence of thrombectomy benefit in patients with NIHSS scores below 10, as seen in BASICS. Together, these two new trials present convincing data that thrombectomy improves outcome in BAO up to 24 h after symptom onset.

Retrospective analyses suggest that better outcome in EVT-treated BAO is associated with earlier treatment,^19^ use of aspiration rather than stent retrievers,^20^ local rather than general anesthesia,^21^ and good collateral supply.^22^ The first pass effect, defined as full recanalization in a single pass of the endovascular thrombectomy device, is a predictor of good outcome for posterior circulation EVT, as it is for the anterior circulation.^23^

### Endovascular treatment of PCA occlusions

For proximal PCA occlusion (P1 or P2 segments), retrospective analyses have shown trends toward better outcomes with EVT versus best medical treatment (which may include IVT).^24^ A similar result was observed in the TOPMOST study evaluating distal PCA occlusions (P2 and P3 segments).^25^ None of these studies showed an increase in symptomatic ICH with EVT.

In conclusion, EVT for posterior circulation stroke is feasible and has similar complication rates to the anterior circulation. It is effective in BAO patients, and the BAOCHE trial showed benefit in some patients up to 24 h. Further RCTs are required to determine effectiveness and which subgroups benefit, in particular in PCA and VA occlusion. IVT does not preclude subsequent endovascular therapy although the benefit of bridging remains unproven in the posterior circulation.

### Imaging to improve patient selection for acute revascularization

Imaging the potential viability of ischemic tissue and collateral patterns could help select patients most likely to respond, and be harmed, from such treatments. The pc-ASPECTS (posterior circulation-Alberta Stroke Program Early CT Score) estimates severe tissue hypoperfusion with non-viability and is ideally assessed on CT-angiography source images.^26^ It independently correlates with clinical outcomes and has also been validated on magnetic resonance imaging diffusion-weighted imaging (MRI-DWI).

Focal hypoperfusion on CT in posterior circulation strokes is associated with poorer outcome independently of other prognostic factors. Severe hypoperfusion on CT within specific areas can be used to construct the Critical Area Perfusion (CAP) score in BAO, correlating well with clinical outcome in EVT patients.^27^ Similarly, the MRI-DWI-based PMT (pons-midbrain and thalamus) score assesses the extent of DWI lesions in these regions in BAO; used in EVT patients, it correlates well with functional outcome.^28^

As opposed to the anterior circulation, no scores exist describing occlusions and/or collaterals encompassing the entire posterior circulation. For BAO, the PC-CS (posterior circulation-collateral score) grading system quantifies occlusions and collateral flow and emphasizes the importance of the posterior communicating arteries. Clinical outcome correlated with the score independently of treatment type.^29^ The BATMAN (Basilar Artery on computed ToMography Angiography) score considers both thrombus burden and collaterals with a lower score associated with poorer outcome.^30^ Such collateral scores are usually based on maximal intensity projections of CTAs which are reconstructed routinely in acute stroke imaging; they are calculated within 2–3 min.

The importance of radiological selection for revascularization in BAO was also shown by the ENDOSTROKE Study Group: in 148 BAO patients undergoing EVT, successful recanalization did not predict outcome on its own, but MRI-based selection was associated with a better outcome.^31^ Similarly, the non-significant results of RCTs in BAO (see above) may require more stringent radiological inclusion criteria for further trials, even in early time windows.

### Decompressive surgery for ischemic and hemorrhagic stroke with mass effect

**Figure 3.**
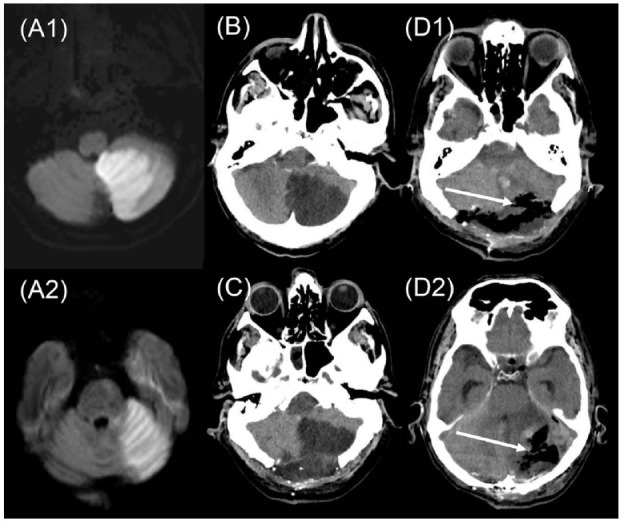
46-year-old man with multilevel posterior circulation stroke and BAO. NIHSS = 8. (a) Diffusion-weighted MRI showing extensive left inferior (A1) and superior (A2) cerebellar infarcts. Basilar artery recanalization by direct thrombectomy 8 h after last proof of good health. (b) Plain CT 5 h later, showing early cerebellar mass effect. (c) Plain CT after decompressive posterior craniectomy of 5 cm diameter, still showing cerebellar mass effect. (d) Plain CT after second craniectomy on day 3 with enlargement of craniectomy diameter to 7 cm and partial resection (arrows) of left inferior(D1) and superior(D2) cerebellar infarcts. Outcome at 3 months, independent, but not working. Copyright Patrik Michel.

Cerebellar stroke, mainly in the inferior territory, may lead to secondary swelling and space-occupying cerebellar infarction. In contrast to decompressive hemicraniectomy for supratentorial stroke, RCTs for this condition are lacking. Expert opinion suggests considering suboccipital decompression in selected patients who deteriorate neurologically from impending brainstem compression.^32,33^ Removal of infarcted cerebellar tissue during this procedure is controversial but may be considered in some situations. An example is shown in Figure 3.

Drainage of cerebrospinal fluid by ventriculostomy should be considered in selected patients in whom the obstructive hydrocephalus is the main cause of neurological deterioration.^32^ It should be accompanied by suboccipital decompressive craniectomy in order to avoid deterioration from upward cerebellar herniation.^29^

In ICH affecting primarily the cerebellum, clinical deterioration can occur quickly due to the narrow anatomical space of the posterior fossa, leading to local mass effect on the brainstem or obstructive hydrocephalus. In such patients, or patients with cerebellar hemorrhages >3 cm in diameter, observational studies suggest better outcome with surgical decompression.^34^ Ventricular catheter insertion alone is not recommended^34^ and may actually be harmful, particularly in patients with compressed cisterns. In contrast to cerebellar hemorrhage, evacuation of brainstem hemorrhages may be harmful and is not recommended.

## Secondary prevention

### Stroke recurrence risk

**Figure 4.**
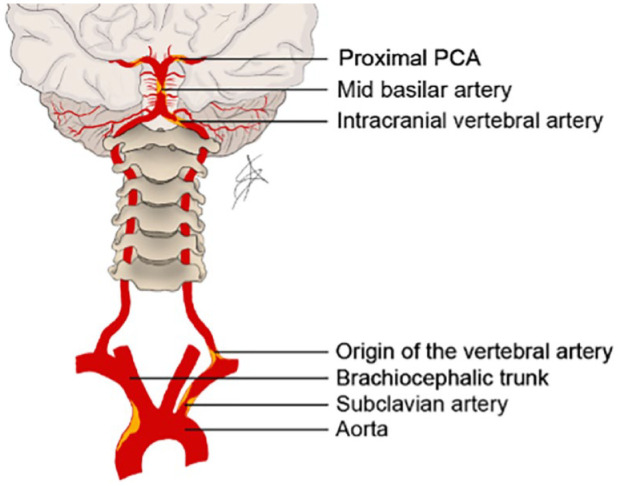
Frequent sites of atherosclerotic plaques in the posterior circulation. PCA: posterior cerebral artery. Drawing Alexander Salerno.

Initially it was suggested posterior circulation TIA and stroke was associated with a lower risk of recurrent stroke than anterior circulation disease.^(3)^ However, prospective natural history studies have shown it is associated with a high risk of early recurrent stroke, particularly in the first few weeks.^35^ The stroke subtype with the highest early recurrent stroke risk is atherosclerotic large artery disease, and the temporal pattern of recurrence is similar to that seen for carotid artery disease.^35^ Typical sites of posterior circulation atherosclerosis are shown in Figure 4. A pooled individual patient analysis of two prospective studies in patients with posterior circulation TIA or stroke, who all had CT- or MR-based angiography to identify stenosis, reported a 90-day recurrent stroke rate for basilar or intracranial vertebral stenosis of 33%, compared with 16% for extracranial vertebral stenosis.^(3)^ This high early recurrence risk suggests posterior circulation large artery stenosis should be treated intensively with antiplatelet agents, as discussed below. It also raises the possibility as to whether revascularization should be performed, in a similar fashion to endarterectomy for symptomatic carotid stenosis.

### Antithrombotic treatment

Medical treatment and risk factor management is similar for both anterior and posterior circulation stroke. We did not identify trials specifically looking at prevention of posterior circulation stroke and therefore our recommendations are based on trials in patients with stroke in all vascular territories. For non-cardioembolic ischemic stroke, clopidogrel (or aspirin) alone is recommended for long-term secondary prevention. However, recent studies randomizing patients to short-term dual antiplatelet therapy with aspirin and clopidogrel immediately after non-cardioembolic stroke (POINT and CHANCE), showed a lower risk of early recurrent stroke compared with aspirin alone.^36^ These included posterior circulation stroke but did not separate treatment effect by vascular territory.

**Figure 5.**
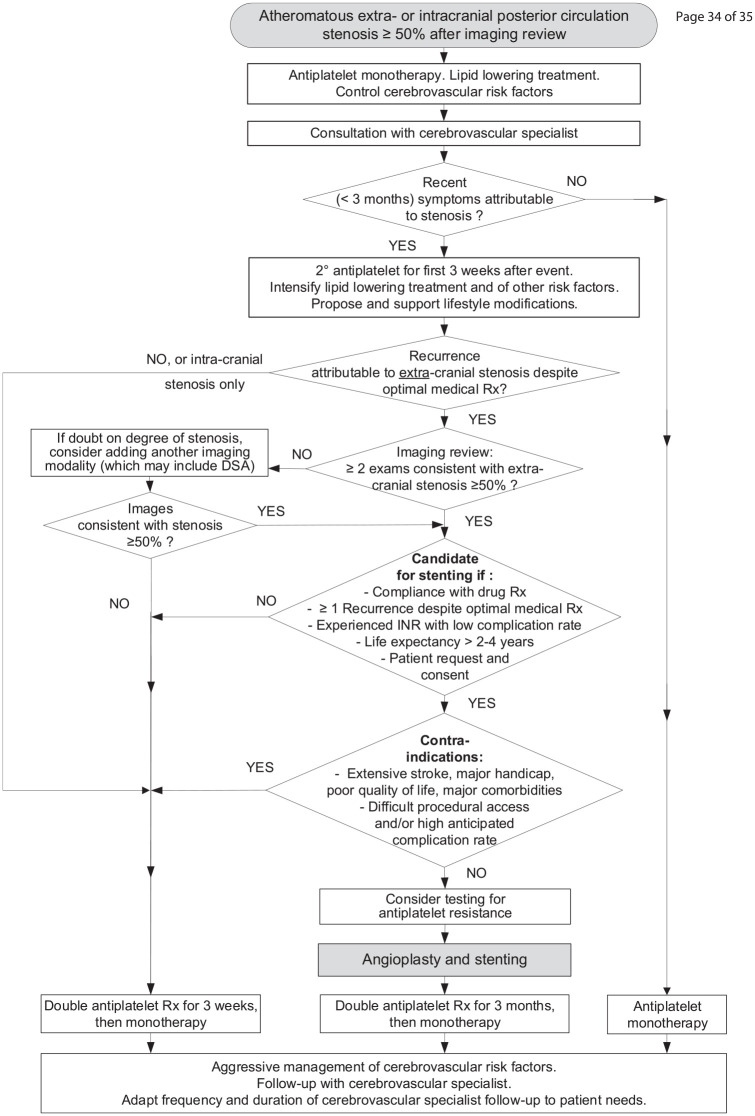
Suggested algorithm for prevention of ischemic strokes stratified by the presence of posterior circulation stenosis. Rx: treatment; DSA: digital subtraction angiography; INR: interventional neuroradiologist.

It is therefore recommended that patients with recent high-risk minor stroke and TIA receive dual antiplatelets (aspirin and clopidogrel or aspirin and ticagrelor).^37^ Analysis of the POINT and CHANCE data suggested benefit of dual antiplatelets for only the first 3 weeks,^36^ so this duration seems reasonable also for the posterior circulation,^37^ before switching to clopidogrel alone. An algorithm for prevention of posterior circulation ischemic strokes is proposed by the authors in Figure 5, based on available data.

Secondary prevention of cardioembolic posterior circulation stroke is as for anterior circulation stroke. Patients with posterior and anterior circulation stroke and atrial fibrillation^38^ appear to have similar risks of ischaemic or hemorrhagic events at 90 days. Whether anticoagulation should be started immediately after ischaemic stroke with atrial fibrillation, or after a period of a week or two, to reduce risks of hemorrhagic transformation is uncertain and being examined in ongoing trials such as OPTIMAS^39^ and ELAN (https://www.clinicaltrials.gov/ct2/show/NCT03148457).

### Control of cerebrovascular risk factors

The medical arm of SAMMPRIS (Stenting versus Aggressive Medical Therapy for Intracranial Arterial Stenosis) emphasized the importance of intensive medical therapy and risk factor control which should be provided to all patients with posterior circulation stenosis.^40^ We provide intensive statin therapy^41^ to all ischemic stroke patients with or without symptomatic stenosis, aiming at low-density lipoprotein cholesterol (LDLC) levels <1.4 mmol/L (<55 mg/L) and a ⩾50% reduction of LDLC when compared to baseline values.^37^ Blood pressure should aim at values below 130/80 mmHg if tolerated.^41,42^ SAMMPRIS and other recent studies also emphasized the importance of lifestyle interventions including smoking cessation, a healthy diet, and physical activity. Secondary prevention for ICH in the anterior and posterior circulations includes lowering of blood pressure to similar values as after ischemic stroke.^34^

### Stenting for symptomatic posterior circulation stenosis

Atheromatous plaques may cause stenosis or occlusion at preferential sites in the vertebral, basilar, and PCAs (Figure 4). These arteries are surgically less accessible than the carotid artery, and therefore, although endarterectomy approaches have been used for vertebral stenosis, they have not been widely adopted.^(3)^ In contrast, neurointerventional angioplasty and stenting techniques are used to treat vertebrobasilar stenoses.

#### Basilar stenosis

The SAMMPRIS trial randomized 451 patients with recently symptomatic anterior and posterior circulation intracranial stenosis to either stenting or intensive medical management including dual antiplatelets with clopidogrel and aspirin.^40^ The 30-day stroke or death rate was 14.7% in the intervention and 5.8% in the medical group. Beyond 30 days, stroke rates were similar between both groups. Almost a quarter of patients (22.5% in the medical and 21.9% in the intervention group) had basilar stenosis. The post-operative risk with stenting was higher than had been expected, while the risk of recurrent stroke in patients on intensive medical therapy, lower than predicted.

In SAMMPRIS, the outcome was not divided according to location of stenosis, but findings from a subsequent analysis demonstrated that basilar artery stenting was associated with a particularly high risk of peri-procedural ischemic stroke (20.8% versus 6.7% for other arteries), consistent with other reports that peri-procedural complications from stenting are particularly high for basilar stenosis. It has been suggested this is because of the complication of disrupting flow in perforating arteries arising directly from the basilar artery.^43^

It has been suggested the Wingspan stents used in SAMMPRIS may have a higher complication rate than other stents. However, the VISSIT (Vitesse Intracranial Stent Study for Ischemic Stroke Therapy) trial, which evaluated a balloon expandable stent in 112 patients with intracranial stenosis, also showed a higher stroke rate in the stenting group; the 30-day primary safety endpoint occurred in 14/58(24%) stented patients compared with 5/53(9.4%) in the medical group.^44^ VISSIT included basilar stenosis, but the primary paper does not give outcome by site of stenosis, and we were unable to obtain this information from the corresponding author.

In conclusion, symptomatic basilar artery stenosis should be treated with intensive medical therapy, and stenting should currently be avoided, mostly because of the risk of perforator artery occlusions.

#### Stenting of the VA

Many studies have shown VA stenting is technically feasible, often with an acceptable complication rate, but these have largely been cases series. Systematic reviews have reported very low complication rates for extracranial vertebral stenosis, and 1% or less for origin stenosis, but higher rates of 5–10% for intracranial vertebral stenosis.^45,46^ Such cases series are open to selection and publication bias, and robust data can only be provided by RCTs.

Five RCTs have assessed effectiveness of angioplasty and stenting in symptomatic vertebral stenosis. Two of these, SAMMPRIS^40^ and VISSIT,^44^ were confined to intracranial stenosis including basilar and intracranial vertebral stenoses. Two more recent trials, VIST (Vertebral Artery Ischemia Trial)^47^ and VAST (Vertebral Artery Stenting Trial),^47^ included only vertebral stenosis, both intracranial and extracranial. One trial, CAVATAS (Carotid and Vertebral Artery Transluminal Angioplasty Study) conducted in the 1990s included predominantly carotid artery stenosis, but also recruited 16 patients with vertebral stenosis.^48^

The largest trial was VIST,^49^ which aimed to recruit 540 patients with ⩾50% symptomatic vertebral stenosis, but recruitment was closed by the funder after 181 patients were enrolled due to recruitment being slower than expected, a decision which in retrospect appears unfortunate. Mean follow-up was 3.5 years. Stenosis was predominantly extracranial (78.7%). The primary endpoint of fatal or non-fatal stroke occurred in five patients in the stented group versus 12 in the medical group (hazard ratio: 0.40, 0.14–1.13, *p* = 0.08). Therefore, although there was an approximately 60% reduction in the rate of recurrent stroke in patients in the stenting arm, this difference was not significant. In a post hoc analysis, when time from randomization was controlled for, which was shorter in the stenting arm, the hazard ratio for the primary endpoint was significant at 0.34 (0.12–0.98, *p* = 0.046). The benefit, if any, appeared higher in patients with extracranial stenosis, in whom the peri-procedural stroke risk was much lower (0 events, versus 2/13 for intracranial stenosis).

VAST^44^ aimed for a sample size of 180 but recruited 115 of which 83% had extracranial stenosis. During mean follow-up of 3 years, there were seven strokes in the medical group and eight strokes in the stenting group. Of the three early strokes, two occurred in nine patients with intracranial stenosis (22%), while only 1 (2%) occurred in 48 patients with extracranial stenosis. The results of VAST were underpowered to detect any treatment difference, but like VIST showed intracranial stenting had a high peri-procedural risk.

**Figure 6.**
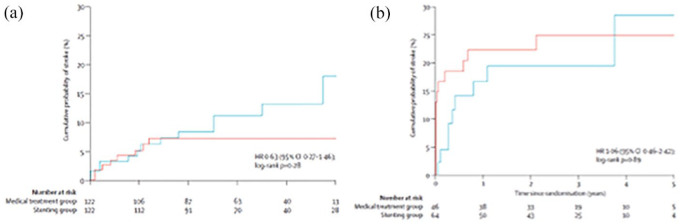
Recurrence rates for any stroke in a preplanned pooled individual patient data analysis of stenting for symptomatic vertebral artery stenosis: (a) Above, stenting for extracranial stenosis; (b) Below, stenting for intracranial stenosis. Blue: no stenting. Red: with stenting.^46^ (reprinted with permission). HR: hazard ratio.

A pooled individual patient data analysis of three of the vertebral stenting trials (VIST, VAST, and SAMMPRIS) was recently conducted.^50^ Across the three trials, 168 participants (46 intracranial, 122 extracranial) were assigned to medical treatment and 186 (64 intracranial, 122 extracranial) to stenting. Peri-procedural risk was higher for intracranial than extracranial stenosis (16% versus 1%, *p* < 0.001). During 1036 person years of follow-up, the hazard ratio for any stroke in the stenting group compared with the medical group was 0.81(0.45–1.44). For extracranial stenosis, it was 0.63 (0.27–1.46), and for intracranial stenosis 1.06 (0.46–2.42). The Kaplan–Meier curves (Figure 6) show stenting for intracranial stenosis was associated with a worse outcome, and the difference persisted for a number of years post-procedure. In contrast, for extracranial stenting, outcomes over the first year were similar between both arms, with a possible divergence benefiting stenting at later time points, although sample size was much reduced at this time.

In conclusion, there is no definitive data from adequately powered RCTs to determine whether stenting for symptomatic vertebral stenosis offers benefit over medical treatment. Available data suggest that with current stenting techniques, intracranial stenosis is associated with a high peri-operative risk, and medical treatment is the preferred option. Whether stenting offers a treatment option for extracranial stenosis remains uncertain. VIST suggested a possible benefit, particularly in patients operated early after symptoms.

For this reason, we may consider angioplasty and stenting as an option in recurrent ischemic events from well-documented extracranial vertebral stenosis, as described in the pragmatic approach described in Figure 5. Further adequately powered trials of stenting in recent symptomatic extracranial vertebral stenosis in comparison with optimal medical therapy are required. In the meantime, aggressive medical treatment of posterior circulation strokes is of paramount importance.

## References

[1] Feigin VL Brainin M Norrving B , et al World Stroke Organization (WSO): Global Stroke Fact Sheet 2022. Int J Stroke 2022; 17: 18–29. 34986727 10.1177/17474930211065917

[2] Salerno A Strambo D Nannoni S Dunet V Michel P . Patterns of ischemic posterior circulation strokes: a clinical, anatomical, and radiological review [published online ahead of print, 2021 Sep 28]. Int J Stroke 2022 17: 714–722. 34581223 10.1177/17474930211046758 PMC9358301

[3] Markus HS van der Worp HB Rothwell PM . Posterior circulation ischaemic stroke and transient ischaemic attack: diagnosis, investigation, and secondary prevention. Lancet Neurol 2013; 12: 989–998. 24050733 10.1016/S1474-4422(13)70211-4

[4] Lindley RI Wardlaw JM Whiteley WN , et al Alteplase for acute ischemic stroke: outcomes by clinically important subgroups in the Third International Stroke Trial. Stroke 2015; 46: 746–756. 25613308 10.1161/STROKEAHA.114.006573

[5] Keselman B Gdovinová Z Jatuzis D , et al Safety and outcomes of intravenous thrombolysis in posterior versus anterior circulation stroke: results from the safe implementation of treatments in stroke registry and meta-analysis. Stroke 2020; 51: 876–882. 31914885 10.1161/STROKEAHA.119.027071

[6] Lee SH Han JH Jung I , et al Do thrombolysis outcomes differ between anterior circulation stroke and posterior circulation stroke? A systematic review and meta-analysis. Int J Stroke 2020; 15: 849–857. 32122288 10.1177/1747493020909634

[7] Sairanen T Strbian D Soinne L , et al Intravenous thrombolysis of basilar artery occlusion: predictors of recanalization and outcome. Stroke 2011; 42: 2175–2179. 21737807 10.1161/STROKEAHA.110.605584

[8] Schonewille WJ Wijman CA Michel P , et al Treatment and outcomes of acute basilar artery occlusion in the Basilar Artery International Cooperation Study (BASICS): a prospective registry study. Lancet Neurol 2009; 8: 724–730. 19577962 10.1016/S1474-4422(09)70173-5

[9] Alemseged F Ng FC Williams C , et al Tenecteplase vs alteplase before endovascular therapy in basilar artery occlusion. Neurology 2021; 96: e1272–e1277. 10.1212/WNL.0000000000011520 33408145

[10] Weber R Minnerup J Nordmeyer H , et al Thrombectomy in posterior circulation stroke: differences in procedures and outcome compared to anterior circulation stroke in the prospective multicentre REVASK registry. Eur J Neurol 2019; 26: 299–305. 30218610 10.1111/ene.13809

[11] Meinel TR Kaesmacher J Chaloulos-Iakovidis P , et al Mechanical thrombectomy for basilar artery occlusion: efficacy, outcomes, and futile recanalization in comparison with the anterior circulation. J Neurointerv Surg 2019; 11: 1174–1180. 31239331 10.1136/neurintsurg-2018-014516 PMC6902072

[12] Alawieh AM Eid M Anadani M , et al Thrombectomy technique predicts outcome in posterior circulation stroke-insights from the STAR collaboration. Neurosurgery 2020; 87: 982–991. 32433730 10.1093/neuros/nyaa179

[13] Writing Group for the BASILAR Group; Zi W Qiu Z , et al Assessment of endovascular treatment for acute basilar artery occlusion via a nationwide prospective registry. JAMA Neurol 2020; 77: 561–573. 32080711 10.1001/jamaneurol.2020.0156 PMC7042866

[14] Liu X Dai Q Ye R , et al Endovascular treatment versus standard medical treatment for vertebrobasilar artery occlusion (BEST): an open-label, randomised controlled trial. Lancet Neurol 2020; 19: 115–122. 31831388 10.1016/S1474-4422(19)30395-3

[15] Langezaal LCM van der Hoeven EJRJ Mont’Alverne FJA , et al Endovascular therapy for stroke due to basilar-artery occlusion. N Engl J Med 2021; 384: 1910–1920. 34010530 10.1056/NEJMoa2030297

[16] Markus HS . The return of in person stroke conferences, thrombectomy for basilar occlusion, and advances in intracerebral haemorrhage. Int J Stroke 2022; 17: 485–486. 35634807 10.1177/17474930221103307

[17] Tao C Li R Zhu Y , et al Endovascular treatment for acute basilar artery occlusion: a multicenter randomized controlled trial (ATTENTION). Int J Stroke 2022; 17: 815–819. 35102797 10.1177/17474930221077164

[18] Li C Wu C Wu L , et al Basilar artery occlusion Chinese endovascular trial: protocol for a prospective randomized controlled study. Int J Stroke 2022; 17: 694–697. 34427475 10.1177/17474930211040923

[19] Greving JP Schonewille WJ Wijman CA , et al Predicting outcome after acute basilar artery occlusion based on admission characteristics. Neurology 2012; 78: 1058–1063. 22442438 10.1212/WNL.0b013e31824e8f40

[20] Bernsen MLE Bruggeman AAE Brouwer J , et al Aspiration versus stent retriever thrombectomy for posterior circulation stroke. Stroke 2022; 53: 749–757. 34666507 10.1161/STROKEAHA.121.034926

[21] Terceño M Silva Y Bashir S , et al Impact of general anesthesia on posterior circulation large vessel occlusions after endovascular thrombectomy. Int J Stroke 2021; 16: 792–797. 33573525 10.1177/1747493020976247

[22] Terceño M Silva Y Bashir S , et al First pass effect in posterior circulation occlusions: analysis from the CICAT registry. Int J Stroke. Epub ahead of print 20 April 2022. DOI: 10.1177/17474930221089772. 35272563

[23] Broocks G Faizy TD Meyer L , et al Posterior circulation collateral flow modifies the effect of thrombectomy on outcome in acute basilar artery occlusion. Int J Stroke 2022; 17: 761–769. 34569885 10.1177/17474930211052262

[24] Strambo D Bartolini B Beaud V , et al Thrombectomy and thrombolysis of isolated posterior cerebral artery occlusion: cognitive, visual, and disability outcomes. Stroke 2020; 51: 254–261. 31718503 10.1161/STROKEAHA.119.026907

[25] Meyer L Stracke CP Jungi N , et al Thrombectomy for primary distal posterior cerebral artery occlusion stroke: the TOPMOST study. JAMA Neurol 2021; 78: 434–444. 33616642 10.1001/jamaneurol.2021.0001 PMC7900924

[26] Puetz V Sylaja PN Coutts SB , et al Extent of hypoattenuation on CT angiography source images predicts functional outcome in patients with basilar artery occlusion. Stroke 2008; 39: 2485–2490. 18617663 10.1161/STROKEAHA.107.511162

[27] Cereda CW Bianco G Mlynash M , et al Perfusion imaging predicts favorable outcomes after basilar artery thrombectomy. Ann Neurol 2022; 91: 23–32. 34786756 10.1002/ana.26272

[28] Liu L Wang M Deng Y , et al Novel diffusion-weighted imaging score showed good prognostic value for acute basilar artery occlusion following endovascular treatment: the pons-midbrain and thalamus score. Stroke 2021; 52: 3989–3997. 34455819 10.1161/STROKEAHA.120.032314

[29] van der Hoeven EJ McVerry F Vos JA , et al Collateral flow predicts outcome after basilar artery occlusion: the posterior circulation collateral score. Int J Stroke 2016; 11: 768–775. 27016515 10.1177/1747493016641951

[30] Alemseged F Shah DG Diomedi M , et al The basilar artery on computed tomography angiography prognostic score for basilar artery occlusion. Stroke 2017; 48: 631–637. 28228577 10.1161/STROKEAHA.116.015492

[31] Singer OC Berkefeld J Nolte CH , et al Mechanical recanalization in basilar artery occlusion: the ENDOSTROKE study. Ann Neurol 2015; 77: 415–424. 25516154 10.1002/ana.24336

[32] Wijdicks EF Sheth KN Carter BS , et al Recommendations for the management of cerebral and cerebellar infarction with swelling: a statement for healthcare professionals from the American Heart Association/American Stroke Association. Stroke 2014; 45: 1222–1238. 24481970 10.1161/01.str.0000441965.15164.d6

[33] van der Worp HB Hofmeijer J Jüttler E , et al European Stroke Organisation (ESO) guidelines on the management of space-occupying brain infarction. Eur Stroke J 2021; 6: III. 10.1177/23969873211027001 PMC8370077 34414304

[34] Hemphill JC Greenberg SM 3rd Anderson CS , et al Guidelines for the management of spontaneous intracerebral hemorrhage: a guideline for healthcare professionals from the American Heart Association/American Stroke Association. Stroke 2015; 46: 2032–2060. 26022637 10.1161/STR.0000000000000069

[35] Gulli G Marquardt L Rothwell PM , et al Stroke risk after posterior circulation stroke/transient ischemic attack and its relationship to site of vertebrobasilar stenosis: pooled data analysis from prospective studies. Stroke 2013; 44: 598–604. 23386676 10.1161/STROKEAHA.112.669929

[36] Pan Y Elm JJ Li H , et al Outcomes associated with clopidogrel-aspirin use in minor stroke or transient ischemic attack: a pooled analysis of clopidogrel in high-risk patients with acute non-disabling cerebrovascular events (CHANCE) and platelet-oriented inhibition in New TIA and minor ischemic stroke (POINT). JAMA Neurol 2019; 76: 1466–1473. 31424481 10.1001/jamaneurol.2019.2531 PMC6704730

[37] Dawson J Merwick Á Webb A , et al European Stroke Organisation expedited recommendation for the use of short-term dual antiplatelet therapy early after minor stroke and high-risk TIA. Eur Stroke J 2021; 6:CLXXXVII–CXCI. 10.1177/23969873211000877 PMC8370083 34414300

[38] Paciaroni M Agnelli G Giustozzi M , et al Timing of initiation of oral anticoagulants in patients with acute ischemic stroke and atrial fibrillation comparing posterior and anterior circulation strokes. Eur Stroke J 2020; 5: 374–383. 33598556 10.1177/2396987320937116 PMC7856592

[39] Best JG Arram L Ahmed N , et al Optimal timing of anticoagulation after acute ischemic stroke with atrial fibrillation (OPTIMAS): protocol for a randomized controlled trial. Int J Stroke 2022; 17: 583–589. 35018878 10.1177/17474930211057722

[40] Derdeyn CP Chimowitz MI Lynn MJ , et al Aggressive medical treatment with or without stenting in high-risk patients with intracranial artery stenosis (SAMMPRIS): the final results of a randomised trial. Lancet 2014; 383: 333–341. 24168957 10.1016/S0140-6736(13)62038-3 PMC3971471

[41] Kleindorfer DO Towfighi A Chaturvedi S , et al 2021 guideline for the prevention of stroke in patients with stroke and transient ischemic attack: a guideline from the American Heart Association/American Stroke Association. Stroke 2021; 52: e364–e467. 34024117 10.1161/STR.0000000000000375

[42] Visseren FLJ Mach F Smulders YM , et al 2021 ESC guidelines on cardiovascular disease prevention in clinical practice. Eur Heart J 2021; 42: 3227–3337. 34458905 10.1093/eurheartj/ehab484

[43] Derdeyn CP Fiorella D Lynn MJ , et al Mechanisms of stroke after intracranial angioplasty and stenting in the SAMMPRIS trial. Neurosurgery 2013; 72: 777–95; discussion795. 10.1227/NEU.0b013e318286fdc8 PMC3696348 23328689

[44] Zaidat OO Fitzsimmons BF Woodward BK , et al Effect of a balloon-expandable intracranial stent vs medical therapy on risk of stroke in patients with symptomatic intracranial stenosis: the VISSIT randomized clinical trial. JAMA 2015; 313: 1240–1248. 25803346 10.1001/jama.2015.1693

[45] Eberhardt O Naegele T Raygrotzki S , et al Stenting of vertebrobasilar arteries in symptomatic atherosclerotic disease and acute occlusion: case series and review of the literature. J Vasc Surg 2006; 43: 1145–1154. 16765230 10.1016/j.jvs.2006.02.027

[46] Stayman AN Nogueira RG Gupta R . A systematic review of stenting and angioplasty of symptomatic extracranial vertebral artery stenosis. Stroke 2011; 42: 2212–2216. 21700936 10.1161/STROKEAHA.110.611459

[47] Compter A van der Worp HB Schonewille WJ , et al Stenting versus medical treatment in patients with symptomatic vertebral artery stenosis: a randomised open-label phase 2 trial. Lancet Neurol 2015; 14: 606–614. 25908089 10.1016/S1474-4422(15)00017-4

[48] Coward LJ McCabe DJ Ederle J , et al Long term outcome after angioplasty and stenting for symptomatic vertebral artery stenosis compared with medical treatment in the carotid and vertebral artery transluminal angioplasty study (CAVATAS): a randomized trial. Stroke 2007; 38: 1526–1530. 17395869 10.1161/STROKEAHA.106.471862

[49] Markus HS Larsson SC Kuker W , et al Stenting for symptomatic vertebral artery stenosis: the vertebral artery ischaemia stenting trial. Neurology 2017; 89: 1229–1236. 28835400 10.1212/WNL.0000000000004385 PMC5606920

[50] Markus HS Harshfield EL Compter A , et al Stenting for symptomatic vertebral artery stenosis: a preplanned pooled individual patient data analysis. Lancet Neurol 2019; 18: 666–673. 31130429 10.1016/S1474-4422(19)30149-8

